# Dalfampridine effects on cognition, fatigue, and dexterity

**DOI:** 10.1002/brb3.559

**Published:** 2016-11-11

**Authors:** Melanie Korsen, Rhina Kunz, Ulf Schminke, Uwe Runge, Thomas Kohlmann, Alexander Dressel

**Affiliations:** ^1^ Department of NeurologyUniversity Medicine GreifswaldGreifswaldGermany; ^2^ Institute of Community MedicineUniversity Medicine GreifswaldGreifswaldGermany; ^3^ Department of NeurologyCarl‐Thiem‐Klinikum CottbusCottbusGermany

**Keywords:** cognition, dalfampridine, dexterity, fatigue, multiple sclerosis, symptomatic therapy, visual evoked potentials

## Abstract

**Objectives:**

Dalfampridine exerts beneficial effects on walking ability in a subgroup of patients with multiple sclerosis (MS). These patients are termed “responders”. Here, we investigated whether the responder status with respect to mobility measures would determine whether dalfampridine treatment exerts a beneficial effect on other MS symptoms. We therefore assessed walking ability, upper limb function, cognition, fatigue, visual evoked potentials (VEPs), depression, and quality of life in patients before and after dalfampridine treatment.

**Methods:**

Patients with MS and impaired mobility were recruited. Maximal walking distance, timed 25 Foot Walk, nine hole peg test, paced auditory serial addition test (PASAT), fatigue severity scale (FSS), VEPs, Beck Depression Inventory (BDI), EuroQol five dimensional questionnaire, and quality of life visual analogue scale were determined before and after 12–14 days of dalfampridine treatment. Repeated measures analysis of variance was applied to determine the effect of dalfampridine treatment.

**Results:**

Of the 34 patients who completed the study, 22 patients were responders and 12 patients nonresponders, according to their performance in mobility measures. Treatment effects for the entire patient cohort were observed for PASAT (*p *= .029) and BDI (*p *= .032). Belonging to the responder cohort did not predict the response to treatment in these tests. For the FSS, response to dalfampridine treatment was dependent on the responder status (*p *= .001) while no effects in the total patient cohort were observed (*p *= .680). Other neurological functions remained unaltered. For VEP latencies, no significant improvements were detected.

**Conclusion:**

In this study, we observed beneficial effects of dalfampridine on cognition, depression, and fatigue. These effects were not limited to patients who responded to dalfampridine with improved mobility measures. These findings underscore the need to assess the beneficial effects of dalfampridine on neurological deficits in MS patients in additional randomized clinical trials.

## 1 Introduction

Multiple sclerosis (MS) is the most frequent cause of nontraumatic disabilities in young adults (Pugliatti et al., 2006). Due to the fact that there is still no curative treatment for MS, available pharmaceuticals aim for a reduction in relapse rate and disability progression or a symptomatic treatment of disease symptoms such as spasticity, ataxia, bladder dysfunction, and neuropathic pain (Chan et al., 2012).

Dalfampridine is the extended‐release formulation of 4‐aminopyridine and the first agent approved for the symptomatic treatment of impaired mobility in MS patients with an expanded disability status scale (EDSS) of 4–7 (European Medicines Agency, 2011). In the two phase III clinical trials, a treatment with 10 mg of dalfampridine twice daily improved the walking ability measured by the timed 25 Foot Walk test (T25FW) in responders by about 25%. Responders accounted for 35–43% of the study population (Goodman et al., 2009, 2010). Nonresponders did not benefit from dalfampridine treatment. The most frequent adverse events were urinary tract infections, insomnia, dizziness, nausea, and headache. Furthermore, dalfampridine might increase the risk of seizure (Goodman et al., 2009, 2010).

Dalfampridine is solely approved for the symptomatic treatment of impaired mobility in MS patients (European Medicines Agency, 2011). Although the compound is a well‐known, broad‐spectrum potassium channel blocker, the exact mechanisms of action leading to the therapeutic effects remain unclear (Dunn & Blight, 2011). However, regarding the assumed effects on conduction, action‐potentials, and synaptic and neuromuscular transmission, it would be very likely that the compound also has an effect on other parts of the central nervous system and therefore on other neurological functions such as vision, fatigue, or cognition (Kim, Goldner, & Sanders, 1980; Shi & Blight, 1997; Smith, Felts, & John, 2000). Indeed, studies investigating whether or not dalfampridine or 4‐aminopyridine exert beneficial effects on other neurological function in MS patients described positive effects on measures of cognitive function, upper limb function, fatigue, and vision (Horton et al., 2013; Jensen, Ravnborg, Mamoei, Dalgas, & Stenager, 2014; Limone, Sidovar, & Coleman, 2013; Magnin et al., 2015; Pavsic, Pelicon, Ledinek, & Sega, 2015; Prugger & Berger, 2013; Rossini et al., 2001; Ruck et al., 2014).

Until today, it remains unclear what distinguishes responders from nonresponders to dalfampridine. We hypothesized that the beneficial effects of dalfampridine on other neurological functions would be observed predominantly in those patients who were responders as defined by mobility scores. We therefore designed a prospective, single center, single arm, observational study of 2 weeks duration to evaluate dalfampridine's effects on other neurological functions in MS patients according to their responder status.

## 2 Methods

### 2.1 Study design

**Figure 1:**
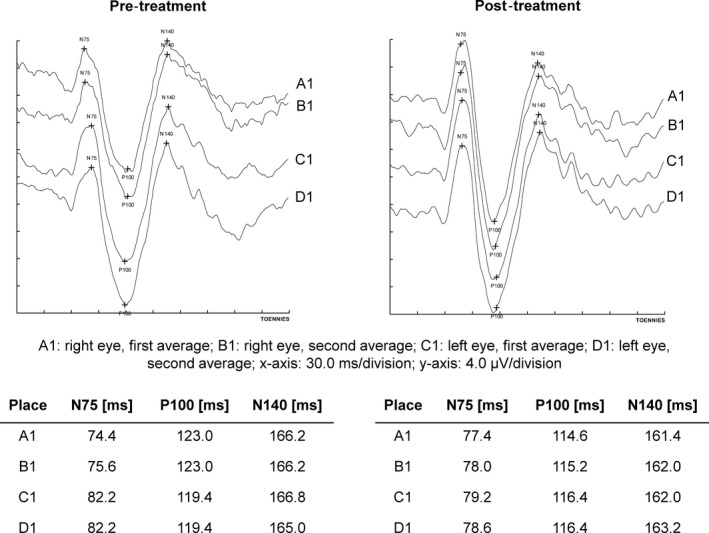
Exemplary VEP recording. Exemplary original VEP recording of a single patient before (pretreatment) and after (posttreatment) two weeks of dalfampridine treatment

Patients were treated with dalfampridine 10 mg twice daily for 12–14 days in a prospective, single center, single arm, observational study. Before treatment initiation and after 12–14 days of treatment, patients underwent detailed physical and neurological examination including EDSS, measurement of the maximal walking distance, T25FW, and the subtests of the multiple sclerosis functional composite (MSFC) (nine hole peg test [9‐HPT], and the paced auditory serial addition test [PASAT]) (Cutter et al., 1999; Fischer, Kniker, Rudick, & Cutter, 2001; Kurtzke, 1983). Electroencephalography (EEG) was performed to detect possible epileptic activity and VEPs to determine the P100 latency. Conventional pattern‐reversal VEPs using checkerboard pattern stimuli (2 reversals per second, large 60 min of arc checks) were measured while patients were in a sitting position in a darkened room. All VEP recordings were performed with the same device (Toennies NeuroScreen plus, E. Jaeger GmbH, Hoechberg, Germany, equipped with a TV monitor) in the same room by an experienced technician with an identical standardized setup throughout the study. Scalp silver–silver chloride cup electrodes were placed according to the international 10/20 system with the recording electrode placed at O_z_ and the reference electrode at *F*p_z_. At least two averages with 100 sweeps were performed to verify reproducibility (Fig. 1). Fatigue, depression, and quality of life were assessed using the fatigue severity scale (FSS), the Beck Depression Inventory (BDI), the EuroQol five dimensional questionnaire (EQ‐5D), and the quality of life visual analogue scale (QolVas) as self‐assessment scales (Krupp, LaRocca, Muir‐Nash, & Steinberg, 1989; Moran & Mohr, 2005; Rabin & de Charro, 2001).

Patients who improved by ≥20% in the T25FW or in the maximum walking distance after 12–14 days of treatment were considered responders in this study. Patients who improved <20% in both tests were defined as nonresponders. VEPs ≥ 120 ms were considered to be pathological.

### 2.2 Patients

All patients included in the study were recruited in the Department of Neurology of the University Medicine Greifswald in 2011 and 2012. Patients were included if they were scheduled to receive dalfampridine treatment due to clinical reasoning, gave written informed consent and met the following inclusion criteria: MS according to the McDonald criteria, age over 18, and an EDSS between 4 and 7 (Polman et al., 2011). Exclusion criteria were a history of epileptic seizure, a creatinine clearance of <80 ml/min calculated according to the Cockcroft‐Gault‐formula and a MS relapse or a change in disease modifying treatment within the preceding 30 days of the study. Furthermore, patients with a comedication that inhibits the OCT2 transporter function (Kido, Matsson, & Giacomini, 2011) were excluded from the study since this is listed as a contraindication in the guidelines of the European Medicines Agency (2011). EEG was performed prior to treatment and after 12–14 days of treatment and patients with any evidence of epileptiform activity in the screening were excluded from the study. The PASAT test is routinely administered in our MS clinic. Therefore, while no formal PASAT training was included in the observational study design, all but five patients had received at least one documented PASAT test before inclusion in this study.

The study was approved by the ethics committee of the Medical Faculty of the University of Greifswald (BB92/11). All patients included in the study gave written, fully informed consent in accordance to the Declaration of Helsinki and a protocol approved by the ethics committee of the University of Greifswald.

### 2.3 Patient selection

**Figure 2:**
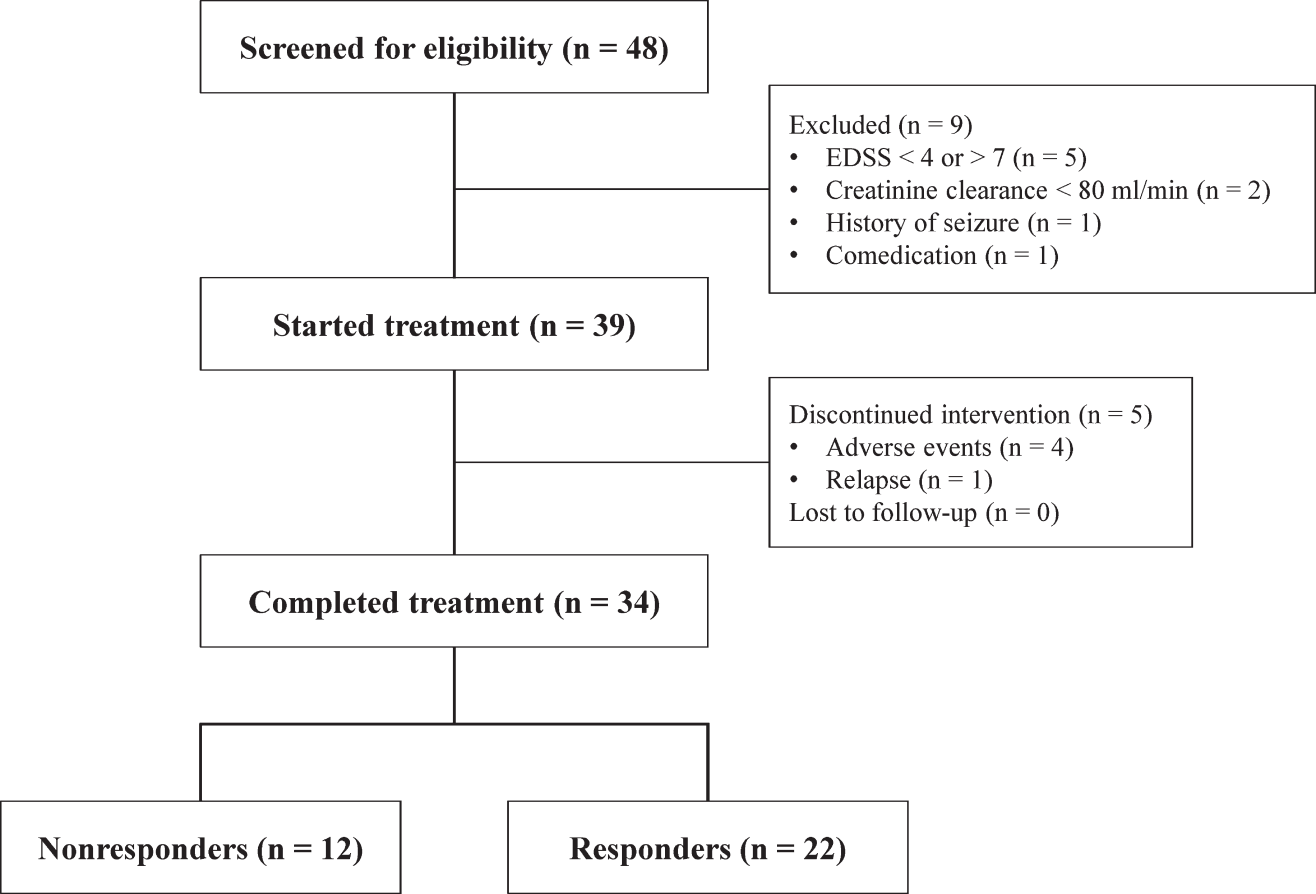
Flowchart of study design and patient assignments

Forty‐eight patients were screened for eligibility. According to the inclusion and exclusion criteria, 39 patients were enrolled in the study and nine patients were excluded (Fig. 2). Five of them did not fulfill the inclusion criteria for the EDSS, two had a creatinine clearance <80 ml/min, one had a history of seizure, and one was taking a comedication that inhibited the OCT2 transporter function. Four patients did not complete the trial due to adverse events (nausea *n *= 2, dizziness *n *= 2, headache *n *= 1, back pain *n *= 1, balance disorder *n *= 1, tremor *n *= 1, asthenia *n *= 1, paresthesia *n *= 1) and one due to a relapse. Thus, a total number of 34 patients were analyzed of which 22 were considered to be responders and 12 to be nonresponders (Fig. 2).

### 2.4 Statistical analysis

The data were analyzed in a per protocol analysis. Patients not completing the treatment period with dalfampridine were followed for adverse events but not included in the analysis. All analyses were carried out using the software IBM SPSS Statistics Ver. 22 by IBM (Armonk, NY USA) or Prism 5.0 by GraphPad Software Inc. (San Diego, CA, USA). For maximal walking distance, T25FW, 9‐HPT, PASAT, FSS, BDI, EQ‐5D, and QuolVas effects of time (pre vs. post) and belonging to the responder cohort were analyzed using repeated measures analysis of variance including effects of time, responder status, and their interaction. For VEPs, effects of eye status (nonaffected vs. affected) and responder status (nonresponder vs. responder) were analyzed using linear mixed models with pre‐post changes in VEPs as the dependent variable, eye and responder status as fixed effects, and a random intercept term accounting for dependencies introduced by simultaneous analysis of right and left eyes of subjects. *p*‐values <.05 were considered statistically significant.

## 3 Results

### 3.1 Demographics and clinical characteristics of nonresponder and responder groups

In this prospective, single center, single arm, observational study, we evaluated the effects of a 12–14 days treatment with dalfampridine in MS patients. Twenty‐two patients were considered to be responders and 12 patients to be nonresponders according to their performance in the maximal walking distance or the T25FW.

**Figure 3:**
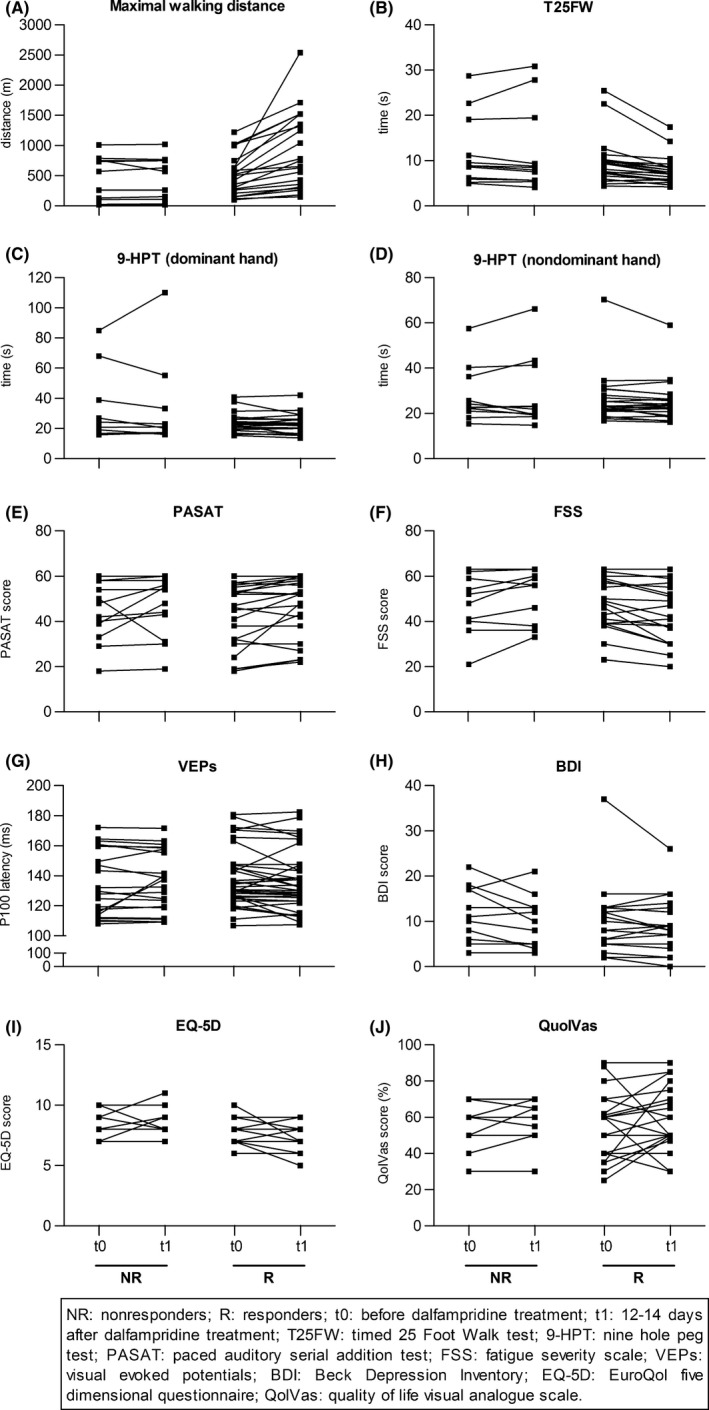
Effects of 2 weeks of dalfampridine treatment in nonresponders and responders. Patients were treated with dalfampridine for 12–14 days. Patients were defined as nonresponders (NR) and responders (R) according to their improvement in maximal walking distance or T25FW (NR < 20%, R ≥ 20%). Before (t0) and after (t1) treatment maximal walking distance (NR:* n *= 12; R: *n *= 22), T25FW (NR:* n *= 12; R: *n *= 22), 9‐HPT (dominant hand, NR:* n *= 10; R: *n *= 22; nondominant hand, NR:* n *= 11; R: *n *= 22), PASAT (NR:* n *= 12; R: *n *= 21), FSS (NR:* n *= 12; R: *n *= 21), visual evoked potentials (VEPs) (NR:* n *= 22, P100 latency measurements of 11 different patients; R: *n *= 38, P100 latency measurements of 20 different patients), BDI (NR:* n *= 11; R: *n *= 22), EQ‐5D (NR:* n *= 11; R: *n *= 22), and QolVas (NR:* n *= 10; R: *n *= 22) were assessed. Data are depicted as absolute values before and after dalfampridine treatment. Statistical analysis are described in the manuscript text

Table 1 summarizes the demographics and clinical characteristics of the responders and nonresponders. Both, responders and nonresponders, had comparable baseline characteristics regarding the maximal walking distance, the T25FW and the other parameters assessed in this study (Table 1). On average, responders improved their maximal walking distance by 387 m or 78% and in the T25FW, by 1.8 s or 18.6%, whereas the performance of nonresponders remained unchanged (Fig. 3A+B). As a direct result of the improved walking function, a decrease in the EDSS by 0.5–1.5 points was observed in 10 of the 22 responders. No patient under dalfampridine treatment developed a clinical seizure, showed epileptic discharges on EEG, or had an altered rhythm in the second EEG.

**Table 1:** Demographics and clinical characteristics of the nonresponder and responder groups

	Nonresponders	Responders	*p*‐value
Number of patients	12	22	
Sex, number of patients (%)			
Male	5 (41.6)	7 (31.8)	
Female	7 (58.3)	15 (68.2)	
Age, mean (*SD*)			
All patients	48.4 (7.8)	48.0 (10.4)	
Male	52.8 (9.7)	45.4 (13.7)	
Female	45.3 (4.8)	49.2 (8.7)	
Disease course, number of patients (%)			
RRMS	5 (41.7)	13 (59.1)	
SPMS	4 (33.3)	8 (36.6)	
PPMS	3 (25.0)	1 (4.5)	
Baseline characteristics, median (interquartile range)			
EDSS	4.5 (4.0–6.5)	4.0 (4.0–5.0)	.2914
Baseline characteristics, mean (*SD*)			
Maximal walking distance (m)	491.9 (357.1)	496.0 (334.2)	.9740
T25FW (s)	11.7 (7.7)	9.5 (5.2)	.3451
9‐HPT, dominant hand (s)	33.2 (24.2)	23.9 (6.3)	.0972
9‐HPT, nondominant hand (s)	27.8 (12.3)	26.2 (10.9)	.7072
PASAT	44.1 (13.0)	42.2 (14.1)	.7052
FSS	50.2 (13.4)	48.8 (11.7)	.7627
P100 latency of VEPs (ms)	134.2 (21.8)	137.7 (19.0)	.5151
BDI	11.8 (6.1)	9.7 (7.5)	.4282
EQ‐5D	8.4 (1.0)	7.8 (1.0)	.1422
QolVas (%)	52.0 (14.8)	56.6 (18.3)	.4876

*SD*, standard deviation; RRMS, relapsing remitting multiple sclerosis; SPMS, secondary progressive multiple sclerosis; PPMS, primary progressive multiple sclerosis; T25FW, timed 25 Foot Walk test; 9‐HPT, nine hole peg test; PASAT, paced auditory serial addition test; FSS, fatigue severity scale; VEPs, visual evoked potentials; BDI, Beck Depression Inventory; EQ‐5D, EuroQol five‐dimensional questionnaire; QolVas, quality of life visual analogue scale.

### 3.2 Upper limb function

Next, we tested whether dalfampridine would also improve dexterity. Therefore, patients undertook the 9‐HPT before and after treatment with dalfampridine. There was no effect of dalfampridine treatment on the performance in the 9‐HPT, neither in the total cohort nor with respect to the responder status (Fig. 3C+D, Table 2).

**Table 2:** Analysis of treatment effects and group effects for main clinical characteristics after 12–14 days of dalfampridine treatment

Clinical characteristics	Patient cohort	Pretreatment, mean (*SD*)	Posttreatment, mean (*SD*)	*p*‐value	*N*
Maximal walking distance (m)	Tx	494.5 (337.0)	742.1 (575.7)	.004	34
	Tx*R	496.0 (334.2)	882.8 (631.6)	.003	22
T25FW (s)	Tx	10.3 (6.1)	9.2 (6.1)	.039	34
	Tx*R	9.5 (5.2)	7.8 (3.1)	.024	22
9‐HPT, dominant hand (s)	Tx	26.8 (14.7)	26.1 (17.4)	.632	32
	Tx*R	23.9 (6.3)	22.9 (6.4)	.744	22
9‐HPT, nondominant hand (s)	Tx	26.7 (11.2)	26.1 (11.5)	.584	33
	Tx*R	26.2 (10.9)	25.0 (9.1)	.194	22
PASAT	Tx	42.9 (13.5)	46.1 (13.3)	.029	33
	Tx*R	42.2 (14.1)	45.8 (13.5)	.651	21
FSS	Tx	49.3 (12.1)	48.1 (13.2)	.680	33
	Tx*R	48.8 (11.7)	45.2 (13.5)	.001	21
BDI	Tx	10.4 (7.0)	9.4 (5.8)	.032	33
	Tx*R	9.7 (7.5)	9.0 (6.0)	.314	22
EQ‐5D	Tx	8.0 (1.0)	7.8 (1.3)	.725	33
	Tx*R	7.8 (1.0)	7.4 (1.2)	.086	22
QolVas (%)	Tx	55.2 (17.2)	58.3 (16.4)	.327	32
	Tx*R	56.6 (18.3)	60.0 (17.2)	.890	22

T25FW, timed 25 Foot Walk test; 9‐HPT, nine hole peg test; PASAT, paced auditory serial addition test; FSS, fatigue severity scale; BDI, Beck Depression Inventory; EQ‐5D, EuroQol five‐dimensional questionnaire; QolVas, quality of life visual analogue scale; Tx, effect of treatment, comparing data from time point 0 (before treatment) to data from time point 1 (12–14 days of dalfampridine treatment). Tx*R, tests whether the group effect (belonging to the responder group) determines the treatment effect; *SD*, standard deviation; *N*, number of included patients.

### 3.3 Cognitive function and fatigue

To evaluate whether dalfampridine may alter the cognitive function of MS patients, we assessed the performance in the PASAT test before and after treatment. Furthermore, we evaluated the severity of fatigue using the FSS questionnaire. After dalfampridine treatment, an overall improvement in the PASAT test could be observed (*p *= .029), whereas the patient's responder status did not determine the change in the PASAT score following treatment (Fig. 3E, Table 2). Furthermore, while there was no overall effect in fatigue score, belonging to the responders group affected the treatment response to dalfampridine in the FSS (*p *= .001) (Fig. 3F, Table 2).

### 3.4 P100 latency in VEPs

To analyze the effects of dalfampridine treatment on the P100 latency, VEPs were performed before and after treatment with dalfampridine. When the entire patient cohort was analyzed, no differences could be observed between baseline characteristics and posttreatment characteristics (Table 3). Since beneficial effects may be limited to patients with demyelination in the optic nerve, we not only analyzed whether the responder status determined the dalfampridine effect but also assessed the effect of eye status (affected eyes with pathological P100 latency ≥ 120 ms before treatment initiation vs. nonaffected eyes with P100 latency <120 ms). While no significant changes could be observed for either group, there was a trend that being a dalfampridine responder or having a delayed P100 latency may result in a beneficial treatment effect (Fig. 3G, Table 3).

**Table 3:** Subanalysis of VEP P100 latencies in dalfampridine‐treated patients with respect to their responder and eye status

Patient cohort	VEP Pre‐Post Change Score	*p*‐value
Overall	−0.65	.526
Responder status		
No	2.42	.116
Yes	−1.58	
Eye status		
Nonaffected	1.31	.222
Affected	−1.77	

VEP Pre‐Post Change Scores show estimated marginal means derived from linear mixed models. Positive (negative) values of change scores denote increase (decrease) of VEP P100 latencies after treatment. Responder status was determined with respect to mobility measures. Eye status indicates whether the P100 latency was pathologically delayed before treatment (affected: ≥ 120 ms; nonaffected: <120 ms). *p*‐values from linear mixed models.

### 3.5 Depression and quality of life

Patients were asked to complete the BDI, EQ‐5D, and QolVas questionnaires before and after treatment with dalfampridine to determine the effect of dalfampridine on depression and quality of life scores. For BDI, an overall beneficial effect of dalfampridine treatment could be observed that was independent of the patient's responder status (*p *= .032) (Fig. 3H, Table 2). There was a trend toward an effect of responder status on the quality of life when assessed by the EQ‐D5, however this was not confirmed in the QolVas (Fig. 3I+J, Table 2).

## 4 Discussion

This study was designed to investigate dalfampridine's effects on several neurological functions in MS patients according to their responder status in mobility measures. For walking ability, a clear dichotomy of patients has been described in earlier studies: Those who improve by more than 20% are commonly considered as dalfampridine responders and those who do not improve, as dalfampridine nonresponders. This concept was already applied to demonstrate beneficial effects in the pivotal phase III clinical trials (Goodman et al., 2009, 2010). Nonetheless, most previous studies that investigated the effect of dalfampridine on other neurological deficits did not make this distinction. This study was performed to test the hypothesis that the responder status with respect to mobility measures would also determine whether other MS symptoms would be positively affected by dalfampridine treatment in these patients.

In our study cohort, 64% of the patients improved in walking ability and are thus considered responders to dalfampridine treatment.

While an open‐label, phase IV study by Macdonell et al. (2016)as well as the studies of Limone et al. (2013) and Pavsic et al. (2015) reported an improvement in quality of life measures in dalfampridine‐treated patients, the improvement in walking ability in our patient cohort was not paralleled by significant improved quality of life scores. This difference may be explained by the fact that the quality of life measures in the study by Limone et al. (2013) increased with time. In agreement with our data, they did not observe any significant changes after two weeks of dalfampridine treatment. This is in line with Macdonell et al. (2016) and Pavsic et al. (2015) who reported quality of life improvements after 4‐ or 12‐week treatment periods. Thus, the 2 weeks treatment and observation period applied in our study may have been too short to identify significant changes in quality of life measures. In line with reports by Pavsic et al. (2015), we observed an effect of dalfampridine on the severity of depression. In our cohort, this effect was independent of the responder status, suggesting that the improved depression score may be independent of improved physical disability.

The data on upper limb function remain contradictory across studies: The patients investigated in this study as well as the patients reported by Goodman et al. (2007) and Ruck et al. (2014) did not improve in their performance in the 9‐HPT following dalfampridine treatment. Other studies, though, have reported improvements in the 9‐HPT in dalfampridine‐treated patients (Allart et al., 2015; Jensen et al., 2014; Lo et al., 2015; Pavsic et al., 2015) while Savin et al. (2016) detected positive effects of dalfampridine treatment only on the dominant hand. Tremor is a common side effect of dalfampridine treatment and may account for the lack of a clinically relevant improvement in the 9‐HPT (European Medicines Agency, 2011).

In line with a number of previous studies, we did observe a beneficial effect of dalfampridine on the cognitive function of MS patients (Jensen et al., 2014; Ruck et al., 2014; Triche, Ruiz, Olson, & Lo, 2016). In our cohort, the improvement of the performance in the PASAT test was independent of the responder status. Furthermore, Magnin et al. (2015) reported increased verbal fluency in dalfampridine responders as well as nonresponders. On the contrary, Pavsic et al. (2015) and Smits et al. (1994) did not show any changes in the PASAT performance of MS patients after dalfampridine or 4‐aminopyridine treatment. All studies that used today's dalfampridine formulation and application scheme were quite small and testing of cognitive function was limited to rather crude tests (PASAT, Symbol Digit Modalities Test or verbal fluency). Future studies addressing the effects of dalfampridine should be designed with lager study cohorts and apply a more detailed neuropsychological assessment.

The strongest evidence to support an effect of 4‐aminopyridine on the severity of fatigue symptoms was derived from a double‐blind, randomized, placebo‐controlled trial by Rossini et al. (2001) who reported a significant improvement of fatigue in patients showing adequate blood concentrations of 4‐aminopyridine. This finding was also supported by subsequent observational studies using dalfampridine (Allart et al., 2015; Pavsic et al., 2015; Ruck et al., 2014; Triche et al., 2016). In our cohort, no significant overall effect of dalfampridine on fatigue was detected but the responder group had a better treatment response than the nonresponders.

It seems obvious that delayed VEP latencies should respond to a treatment aimed at increasing conduction velocity in demyelinated axons. Unexpectedly, the data reported in previous studies remain contradictory. While Ruck et al. (2014) did not observe any beneficial effects after dalfampridine treatment, three additional studies reported improvements after 4‐aminopyridine treatment in MS patients (Davis, Stefoski, & Rush, 1990; van Diemen et al., 1993; Horton et al., 2013). In our patient cohort, no beneficial effect of dalfampridine treatment was detected in the overall cohort. Nevertheless, there was a trend indicating that a treatment response could depend on having a pathological P100 latency or belonging to the responder group. More detailed studies are warranted to investigate dalfampridine effects on the latencies of evoked potentials.

We are aware of the limitations of this study; the study is prospective but observational. Therefore, patients were eligible for the study due to their impaired walking ability and not based on other neurological deficits. Thus, beneficial effects may have been missed when the overall performance was not significantly impaired. This problem is highlighted by the equivocal data with respect to the effect of dalfampridine on P100 latencies.

In summary, our data could not provide evidence for the hypothesis that beneficial effects of dalfampridine on additional neurological functions may be restricted to the subgroup of patients that is defined as treatment responders according to their walking abilities. However, this study supports previous results suggesting that complex neurological deficits, including fatigue and cognition, can be positively affected by dalfampridine.

Larger interventional randomized studies that fulfill the criteria to generate class I evidence are warranted.

## Sources of Funding Statement

This work was supported by in‐house funding. Melanie Korsen was supported by scholarships from the German National Academic Foundation and the DOMAGK program of the Medical Faculty of the University Greifswald.

## Conflict of Interest Statement

Melanie Korsen, Rhina Kunz, Uwe Runge, and Thomas Kohlmann declare no conflict of interest. Ulf Schminke's research activities were funded by the National Institute of Neurological Disorders and Stroke (subaward A08580 M10A10647), the Federal Ministry of Education and Research, Germany (grant 03IS2061A), the Federal State of Mecklenburg‐West Pomerania, Germany, and Siemens Healthcare, Erlangen, Germany; and he received speaker's honoraria from the German Society of Clinical Neurophysiology and from the Ultrasound Academy of the German Society of Ultrasound in Medicine. Alexander Dressel has received funding for research projects, travel grants, and speaker honoraria from Bayer Schering, Biogen‐Idec, Genzyme, Merck Serono, Novartis and Teva.

## References

[brb3559-bib-0001] Allart, E. , Benoit, A. , Blanchard‐Dauphin, A. , Tiffreau, V. , Thevenon, A. , Zephir, H. , … Vermersch, P. (2015). Sustained‐released fampridine in multiple sclerosis: effects on gait parameters, arm function, fatigue, and quality of life. Journal of Neurology, 262, 1936–1945. 26041616 10.1007/s00415-015-7797-1

[brb3559-bib-0002] Chan, A. , Flachenecker, P. , Gold, R. , Haghikia, A. , Hellwig, K. , Hemmer, B. , … Zipp, F. (2012). DGN/KKNMS Leitlinie zur Diagnose und Therapie der MS. Retrieved from http://wwwdgnorg/images/stories/dgn/leitlinien/LL_MS_Neu/DGN-KKNMS_MS-LL_20120809_frei_neu4pdf11012013.

[brb3559-bib-0003] Cutter, G. R. , Baier, M. L. , Rudick, R. A. , Cookfair, D. L. , Fischer, J. S. , Petkau, J. , … Willoughby, E. (1999). Development of a multiple sclerosis functional composite as a clinical trial outcome measure. Brain, 122, 871–882. 10355672 10.1093/brain/122.5.871

[brb3559-bib-0004] Davis, F. A. , Stefoski, D. , & Rush, J. (1990). Orally administered 4‐aminopyridine improves clinical signs in multiple sclerosis. Annals of Neurology, 27, 186–192. 2317014 10.1002/ana.410270215

[brb3559-bib-0005] van Diemen, H. A. , Polman, C. H. , van Dongen, M. M. , Nauta, J. J. , Strijers, R. L. , van Loenen, A. C. , … Koetsier, J. C. (1993). 4‐Aminopyridine induces functional improvement in multiple sclerosis patients: a neurophysiological study. Journal of the Neurological Sciences, 116, 220–226. 8336169 10.1016/0022-510x(93)90329-w

[brb3559-bib-0006] Dunn, J. , & Blight, A. (2011). Dalfampridine: a brief review of its mechanism of action and efficacy as a treatment to improve walking in patients with multiple sclerosis. Current Medical Research and Opinion., 27, 1415–1423. 21595605 10.1185/03007995.2011.583229

[brb3559-bib-0007] EuropeanMedicinesAgency . (2011). European public assessment report (EPAR) for Fampyra. Retrieved from http://wwwemaeuropaeu/docs/en_GB/document_library/EPAR_-_Product_Information/human/002097/WC500109956pdf25102014.

[brb3559-bib-0008] Fischer, J. S. , Jak, A. J. , Kniker, J. E. , Rudick, R. A. , & Cutter, G. (2001). Multiple Sclerosis Functional Composite (MSFC): Administration and scoring manual. New York, NY: National Multiple Sclerosis Society.

[brb3559-bib-0009] Goodman, A. D. , Brown, T. R. , Edwards, K. R. , Krupp, L. B. , Schapiro, R. T. , Cohen, R. , … Blight, A. R. (2010). A phase 3 trial of extended release oral dalfampridine in multiple sclerosis. Annals of Neurology, 68, 494–502. 20976768 10.1002/ana.22240

[brb3559-bib-0010] Goodman, A. D. , Brown, T. R. , Krupp, L. B. , Schapiro, R. T. , Schwid, S. R. , Cohen, R. , … Blight, A. R. (2009). Sustained‐release oral fampridine in multiple sclerosis: a randomised, double‐blind, controlled trial. Lancet, 373, 732–738. 19249634 10.1016/S0140-6736(09)60442-6

[brb3559-bib-0011] Goodman, A. D. , Cohen, J. A. , Cross, A. , Vollmer, T. , Rizzo, M. , Cohen, R. , … Blight, A. R. (2007). Fampridine‐SR in multiple sclerosis: a randomized, double‐blind, placebo‐controlled, dose‐ranging study. Multiple Sclerosis, 13, 357–368. 17439905 10.1177/1352458506069538

[brb3559-bib-0012] Horton, L. , Conger, A. , Conger, D. , Remington, G. , Frohman, T. , Frohman, E. , & Greenberg, B. (2013). Effect of 4‐aminopyridine on vision in multiple sclerosis patients with optic neuropathy. Neurology, 80, 1862–1866. 23616154 10.1212/WNL.0b013e3182929fd5 PMC3908347

[brb3559-bib-0013] Jensen, H. , Ravnborg, M. , Mamoei, S. , Dalgas, U. , & Stenager, E. (2014). Changes in cognition, arm function and lower body function after slow‐release Fampridine treatment. Multiple Sclerosis, 20, 1872–1880. 24852920 10.1177/1352458514533844

[brb3559-bib-0014] Kido, Y. , Matsson, P. , & Giacomini, K. M. (2011). Profiling of a prescription drug library for potential renal drug‐drug interactions mediated by the organic cation transporter 2. Journal of Medicinal Chemistry, 54, 4548–4558. 21599003 10.1021/jm2001629 PMC3257218

[brb3559-bib-0015] Kim, Y. I. , Goldner, M. M. , & Sanders, D. B. (1980). Facilitatory effects of 4‐aminopyridine on normal neuromuscular transmission. Muscle and Nerve, 3, 105–111. 6245354 10.1002/mus.880030202

[brb3559-bib-0016] Krupp, L. B. , LaRocca, N. G. , Muir‐Nash, J. , & Steinberg, A. D. (1989). The fatigue severity scale. Application to patients with multiple sclerosis and systemic lupus erythematosus. Archives of Neurology, 46, 1121–1123. 2803071 10.1001/archneur.1989.00520460115022

[brb3559-bib-0017] Kurtzke, J. F. (1983). Rating neurologic impairment in multiple sclerosis: an expanded disability status scale (EDSS). Neurology, 33, 1444–1452. 6685237 10.1212/wnl.33.11.1444

[brb3559-bib-0018] Limone, B. L. , Sidovar, M. F. , & Coleman, C. I. (2013). Estimation of the effect of dalfampridine‐ER on health utility by mapping the MSWS‐12 to the EQ‐5D in multiple sclerosis patients. Health and Quality of Life Outcomes, 11, 105. 23799913 10.1186/1477-7525-11-105 PMC3699372

[brb3559-bib-0019] Lo, A. C. , Ruiz, J. A. , Koenig, C. M. , Anderson, B. M. , Olson, K. M. , & Triche, E. W. (2015). Effects of dalfampridine on multi‐dimensional aspects of gait and dexterity in multiple sclerosis among timed walk responders and non‐responders. Journal of the Neurological Sciences, 356, 77–82. 26139339 10.1016/j.jns.2015.06.008

[brb3559-bib-0020] Macdonell, R. , Nagels, G. , Laplaud, D. A. , Pozzilli, C. , de Jong, B. , Martins da Silva, A. , … Soelberg Sorensen, P. (2016). Improved patient‐reported health impact of multiple sclerosis: The ENABLE study of PR‐fampridine. Multiple Sclerosis, 22, 944–954. 26447066 10.1177/1352458515606809

[brb3559-bib-0021] Magnin, E. , Sagawa, Y. Jr , Chamard, L. , Berger, E. , Moulin, T. , & Decavel, P. (2015). Verbal fluencies and fampridine treatment in multiple sclerosis. European Neurology, 74, 243–250. 26624899 10.1159/000442348

[brb3559-bib-0022] Moran, P. J. , & Mohr, D. C. (2005). The validity of Beck Depression Inventory and Hamilton Rating Scale for Depression items in the assessment of depression among patients with multiple sclerosis. Journal of Behavioral Medicine, 28, 35–41. 15887874 10.1007/s10865-005-2561-0

[brb3559-bib-0023] Pavsic, K. , Pelicon, K. , Ledinek, A. H. , & Sega, S. (2015). Short‐term impact of fampridine on motor and cognitive functions, mood and quality of life among multiple sclerosis patients. Clinical Neurology and Neurosurgery, 139, 35–40. 26363365 10.1016/j.clineuro.2015.08.023

[brb3559-bib-0024] Polman, C. H. , Reingold, S. C. , Banwell, B. , Clanet, M. , Cohen, J. A. , Filippi, M. , … Wolinsky, J. S. (2011). Diagnostic criteria for multiple sclerosis: 2010 revisions to the McDonald criteria. Annals of Neurology, 69, 292–302. 21387374 10.1002/ana.22366 PMC3084507

[brb3559-bib-0025] Prugger, M. , & Berger, T. (2013). Assessing the long‐term clinical benefit of prolonged‐release fampridine tablets in a real‐world setting: a review of 67 cases. Patient Related Outcome Measures, 4, 75–85. 24187513 10.2147/PROM.S42957 PMC3810492

[brb3559-bib-0026] Pugliatti, M. , Rosati, G. , Carton, H. , Riise, T. , Drulovic, J. , Vecsei, L. , & Milanov, I. (2006). The epidemiology of multiple sclerosis in Europe. European Journal of Neurology, 13, 700–722. 16834700 10.1111/j.1468-1331.2006.01342.x

[brb3559-bib-0027] Rabin, R. , & de Charro, F. (2001). EQ‐5D: a measure of health status from the EuroQol Group. Annals of Medicine, 33, 337–343. 11491192 10.3109/07853890109002087

[brb3559-bib-0028] Rossini, P. M. , Pasqualetti, P. , Pozzilli, C. , Grasso, M. G. , Millefiorini, E. , Graceffa, A. , … Caltagirone, C. (2001). Fatigue in progressive multiple sclerosis: results of a randomized, double‐blind, placebo‐controlled, crossover trial of oral 4‐aminopyridine. Multiple Sclerosis, 7, 354–358. 11795455 10.1177/135245850100700602

[brb3559-bib-0029] Ruck, T. , Bittner, S. , Simon, O. J. , Gobel, K. , Wiendl, H. , Schilling, M. , & Meuth, S. G. (2014). Long‐term effects of dalfampridine in patients with multiple sclerosis. Journal of the Neurological Sciences, 337, 18–24. 24290498 10.1016/j.jns.2013.11.011

[brb3559-bib-0030] Savin, Z. , Lejbkowicz, I. , Glass‐Marmor, L. , Lavi, I. , Rosenblum, S. , & Miller, A. (2016). Effect of Fampridine‐PR (prolonged released 4‐aminopyridine) on the manual functions of patients with Multiple Sclerosis. Journal of the Neurological Sciences, 360, 102–109. 26723984 10.1016/j.jns.2015.11.035

[brb3559-bib-0031] Shi, R. , & Blight, A. R. (1997). Differential effects of low and high concentrations of 4‐aminopyridine on axonal conduction in normal and injured spinal cord. Neuroscience, 77, 553–562. 9472411 10.1016/s0306-4522(96)00477-0

[brb3559-bib-0032] Smith, K. J. , Felts, P. A. , & John, G. R. (2000). Effects of 4‐aminopyridine on demyelinated axons, synapses and muscle tension. Brain, 123(Pt 1), 171–184. 10611131 10.1093/brain/123.1.171

[brb3559-bib-0033] Smits, R. C. , Emmen, H. H. , Bertelsmann, F. W. , Kulig, B. M. , van Loenen, A. C. , & Polman, C. H. (1994). The effects of 4‐aminopyridine on cognitive function in patients with multiple sclerosis: a pilot study. Neurology, 44, 1701–1705. 7936300 10.1212/wnl.44.9.1701

[brb3559-bib-0034] Triche, E. W. , Ruiz, J. A. , Olson, K. M. , & Lo, A. C. (2016). Changes in cognitive processing speed, mood, and fatigue in an observational study of persons with multiple sclerosis treated with Dalfampridine‐ER. Clinical Neuropharmacology, 39, 73–80. 26818040 10.1097/WNF.0000000000000130

